# Sweet/Dessert Foods Are More Appealing to Adolescents after Sleep Restriction

**DOI:** 10.1371/journal.pone.0115434

**Published:** 2015-02-23

**Authors:** Stacey L. Simon, Julie Field, Lauren E. Miller, Mark DiFrancesco, Dean W. Beebe

**Affiliations:** 1 Children’s Hospital Colorado & University of Colorado School of Medicine, Aurora, CO, United States of America; 2 Cincinnati Children’s Hospital Medical Center, Cincinnati, OH, United States of America; 3 Department of Psychology, University of Connecticut, Storrs, CT, United States of America; 4 Department of Pediatrics, University of Cincinnati College of Medicine, Cincinnati, OH, United States of America; Simon Fraser University, CANADA

## Abstract

**Study Objective:**

Examine the effect of experimental sleep restriction (SR) on adolescents’ subjective hunger and perceived appeal of sweet/dessert foods versus other foods. A secondary goal was to replicate previous findings on the effects of SR on dietary intake.

**Design:**

Randomized cross-over sleep restriction-extension paradigm.

**Setting:**

Sleep was obtained and monitored at home. Outcome measures were gathered during office visits.

**Participants:**

31 typically-developing adolescents aged 14–17 years.

**Interventions:**

The three-week protocol consisted of a baseline week, followed randomly by five consecutive nights of SR (6.5 hours in bed) versus healthy sleep duration (HS; 10 hours in bed), a 2-night wash-out period, and a 5-night cross-over.

**Measurements:**

Sleep was monitored via actigraphy. The morning after each experimental condition, teens rated their hunger, underwent a 24-hour diet recall interview, and rated the appeal of a series of pictures of sweet/dessert foods (e.g., ice cream, candy) and non-sweets (meat, eggs, fruits, vegetables).

**Results:**

Teens rated pictures of sweet/dessert foods to be more appealing after SR than after HS (Cohen’s *d* = .41, t = 2.07, *p* = .045). The sleep manipulation did not affect self-reported hunger or the appeal of non-sweet foods (*p* >.10). Consistent with our prior work, intake of overall calories was 11% higher and consumption of sweet/dessert servings was 52% greater during SR than HS.

**Conclusions:**

Adolescent SR appears to increase the subjective appeal of sweet/dessert foods, indicating a potential mechanism by which SR might contribute to weight gain and the risk for obesity and chronic illness.

## Introduction

Short sleep correlates with both concurrent and future obesity, leading to speculation that inadequate sleep may contribute to the obesity crisis [1,2]. Indeed, experimentally-induced acute sleep deprivation increases adults’ caloric intake beyond the increased energy required to maintain wakefulness [3,4]. We recently reported similar findings in adolescents (see S1 PDF), who ate significantly more foods high in simple sugars, especially sweets and desserts, when sleep-restricted (SR) than when well-rested [5]. While studying this phenomenon is important in any age range, we consider it particularly important during *adolescence* because this developmental period is uniquely characterized by increased exposure to SR [6] at the same time that individuals’ dietary choices become more independent and form the foundation for enduring dietary patterns [7].

Additional work is needed to replicate our findings and to explicate the mechanism by which SR might affect adolescents’ dietary behaviors. One potential mechanism is a shift in hedonic value. Using behavioral economics theory, if SR causes teens to perceive sweet-tasting foods as more appealing, the intake of those foods would be expected to increase [8]. Sleep-deprived adults experience an increase in subjective cravings for calorie-dense foods.[9] However, it is difficult to generalize adult findings to adolescents because of developmental differences in sleep and dietary needs.

Here we report on a follow-up study in which we examined changes in subjective hunger and perceived appeal of sweets/desserts and non-sweet food in a new sample of adolescents after 5 nights of experimental SR versus healthy-sleep duration (HS). We hypothesized that teens would report greater hunger and rate sweets/desserts, but not non-sweets, as more appealing after SR than after HS. Finally, we explored whether the effect sizes for intake of overall calories and sweets/desserts in the current sample were similar to those in our previous study.

## Methods

### Ethics Statement

All procedures were approved by the Institutional Review Board at Cincinnati Children’s Hospital Medical Center. All participants and their parents provided written documentation of informed assent and consent following receipt of print and verbal information regarding the study.

### Participants

Participants were recruited from flyers posted throughout a regional care network and, to help ensure representation of underserved populations, research staff were available to answer questions in an adolescent health clinic that tends to serve low-income families. Interested adolescents and their parent were contacted via phone for formal screening. Similar to our prior study [5], we enrolled typically-developing 14–17 year-olds without a current psychiatric diagnosis or history of neurological illness/injury. In addition, we excluded for intellectual disability or marked obesity (IQ<70 or BMI>30, initially screened by parent and teen report, then confirmed objectively during a baseline visit).

### Sleep Manipulation and Assessment

**Fig 1:**
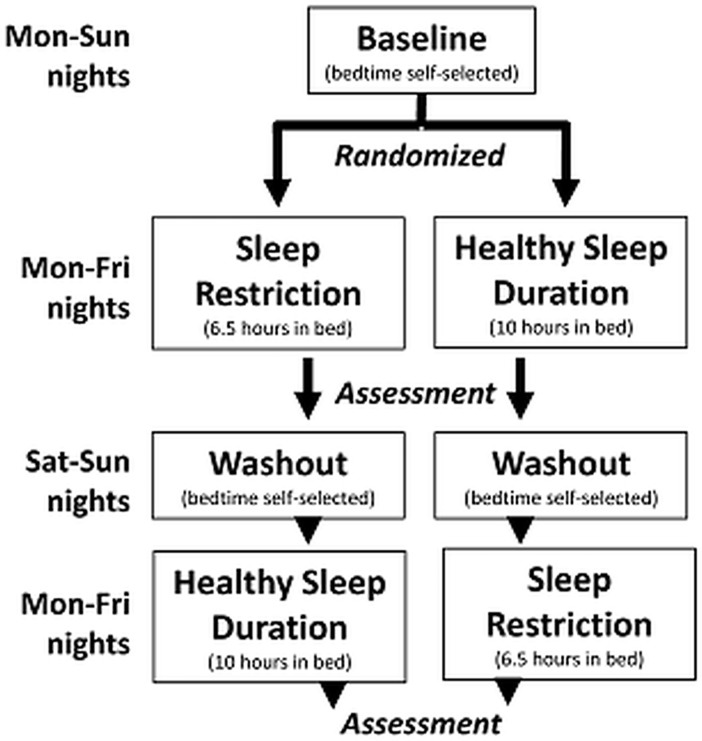
Schematic Diagram of Sleep Protocol. Rise-time for all weeks was determined by when teens needed to awaken to arrive at the study location by 8:30am. Teens self-selected bedtime during the baseline week and Saturday/Sunday nights between conditions. During the experimental weeks, bedtimes Monday-Friday were altered to allow sleep opportunity of 6.5-hours (SR) versus 10-hours (HS).

The 3-week sleep protocol was identical to the prior study (Fig. 1). Teens’ circadian phase was first stabilized in a baseline week, during which they could self-select their bedtimes, but were asked to consistently awaken at a time that would allow them to come in to our office for an 8:30 AM appointment/visit. That wake time was maintained throughout the study (i.e., subjects were not allowed to “sleep in” any morning during the study, including the weekends) to minimize shifts in circadian rhythm. Adolescents who successfully completed the baseline week were randomized to an order of the experimental conditions (SR first vs. HS first) in a counterbalanced, within-subjects crossover design. When in the SR condition, teens’ bedtimes were adjusted to allow 6.5 hours in bed each night for 5 nights. When in the HS condition, their bedtimes were set to allow 10 hours in bed each night for 5 nights. The conditions were separated by a 2-night washout with the same sleep instructions as the baseline. Teens were asked to refrain from napping and limit caffeine intake to no more than 1 coffee or energy drink per day or no more than 2 caffeinated sodas per day.

All sleep occurred at home and was monitored via a daily sleep diary and actigraphy (MicroMotionlogger Sleep Watch, Ambulatory Monitoring, Inc.). Assessments were conducted on Saturday mornings after each experimental condition. Actigraphy data were uploaded and reviewed with both teen and parent during each visit to verify accuracy. Artifact-free actigraphy data were run though a validated algorithm [10] to determine each teen’s average sleep duration within each sleep condition.

### Outcome Measures

The primary outcomes for this study were obtained via a computerized food-appeal rating-system. Two matched sets with 84 photos of 42 sweets/desserts and 42 non-sweets (fruits/vegetables, meat/eggs) were compiled. One set was viewed at each experimental week visit and teens were asked to rate how appetizing each picture looked on a 1–4 scale (“gross,” “OK,” “good,” “delicious”). Internal consistency of ratings was strong (sweets α = .90-.92, non-sweets α = .86-.91). Each teen’s mean rating was computed for each food type (sweets, non-sweets) within each condition (SR and HS), yielding composites with high test-retest reliability (*r* = .79-.83). Teens rated their hunger on a 4-point scale from “not hungry” to “very hungry” immediately after the food-appeal ratings.

To allow for exploratory comparison against our prior dietary findings, roughly 3/4 of the sample also underwent a 24-hour diet recall at each experimental week assessment visit using the USDA Multiple Pass Method [11]. Missing data were due to unavailability of trained interviewers at the beginning of data collection. All dietary interviews were conducted between 8:30 and 11:30 am, with the interviews staggered such that any given teen was interviewed at the same time of day across both experimental conditions. There was no apparent relationship between time of the interview and our dietary outcomes, probably because all interviews were conducted during a narrow time window, and asked about dietary intake the previous day (i.e., interviews conducted Saturday morning asked about food intake Friday). Using previously-described methods [5], total caloric intake and number of servings of sweets/desserts consumed during each assessed 24-hour period were calculated.

### Statistical Analyses

For the food appeal analyses, subjects who indicated a strong dislike (mean rating <2) of a particular food type were removed from that analysis. For all outcome variables, potential carryover effects of the sleep manipulation were explored via general linear modeling, entering as predictors the sleep condition, order in which the sleep conditions were presented, and the order X condition interaction. A significant interaction would indicate a carryover effect. No significant interaction was found across any of our outcome measures (all p > .05), indicating that the effect of sleep manipulation did not vary based on the order in which HS or SR was presented. This allowed us to simplify analyses to paired sample t-tests that examined the effects of sleep manipulation on food appeal and hunger ratings, as well as on sweet/dessert and caloric intake.

## Results

### Sample

Of 38 teens randomized to the sleep manipulation, one dropped out and 6 were non-adherent to the sleep protocol (defined as <60 minutes difference in average sleep duration across the SR and HS conditions), leaving a final sample of 31 (59% female, 52% Caucasian, 41% African-American, mean age 15.7 ± 1.0 years). Actigraphs malfunctioned for 3 subjects, whose adherence to the sleep regimen was confirmed by parent report. The nightly sleep for the other 28 was markedly shorter during SR (6.7 ± 0.6 hr) than HS (9.1 ± 0.7 hr) (p<.0001). As planned, this difference in sleep duration was due to later sleep onset during SR (after midnight: 0:34 ± 0:45) than HS (22:06 ± 0:57) (p<.0001), in the presence of highly similar wake times across conditions (SR: 7:12 ± 0:25 vs. HS: 7:11 ± 0:33) (p > .70).

### Food Appeal and Hunger

**Fig 2:**
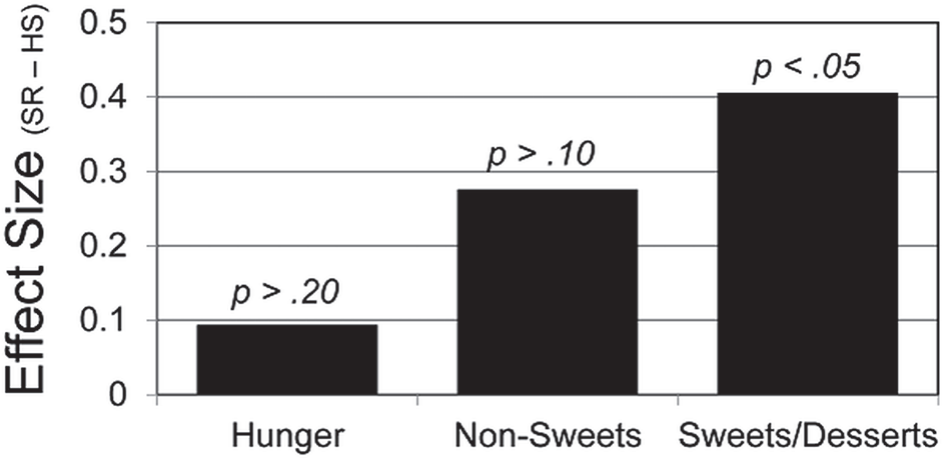
Effect of Sleep Restriction on Adolescents’ Ratings of Hunger and the Appeal of Sweet/Dessert Foods and Non-Sweet Foods (meats, eggs, fruits, vegetables). To promote comparison across outcome measures, effects are expressed as the standardized Cohen’s *d*. Cohen’s *d* represents the average of subjects’ differences in ratings across the two experimental sleep conditions, divided by the standard deviation of those differences. By convention, *d* = .20 indicates a small effect and *d* = .50 indicates a medium-sized effect [21]. Positive values indicate higher ratings in the Sleep Restriction (SR) condition than in the Healthy Sleep Duration (HS) condition.

Fig. 2 summarizes effects of the sleep manipulation on food appeal and hunger ratings. Images of sweets/desserts were rated more appealing after SR than HS (*t* = 2.07, *p* = .049), but the effect of sleep manipulation was non-significant for self-reported hunger and the appeal of non-sweet foods (*p* >.10). In exploratory analyses, results were similarly non-significant when the non-sweets were split into meats/eggs versus fruits/vegetables.

### Sweet/Dessert Foods and Calorie Consumption

In our prior work, after removing outliers who consumed >4000 calories/day, SR increased caloric intake by 9% and intake of sweets/desserts by 130%. Using the same criteria, the 23 teens in this study with dietary data ate 11% more calories (kcal) during SR (1860 ± 689 kcal) than HS (1647 ± 508 kcal) and 52% more servings of sweets/desserts during SR (0.74 ± 1.49 servings) than HS (0.49 ± 0.76 servings). Larger effects were observed without removing caloric outliers (SR increased caloric intake by 23% and intake of sweets/desserts by 73%), but it is difficult to compare these findings to our previous publication due to cross-sample differences in selection criteria. Applying our previously-published selection criteria to pool data from both studies (n = 68), teens consumed 10% more calories in a 24-hour period during SR (1934 ± 740 kcal) than HS (1764 ± 649 kcal), *d* = .25, *t* = 2.1 (*p* = .039), and 110% more servings of sweets/desserts during SR (1.46 ± 2.11 servings) than HS (0.75 ± 1.13 servings), *d* = .30, *t* = 2.4 (*p* = .017).

## Discussion

This study builds upon our prior work in three ways. First, it substantively replicated in a new sample our prior finding that SR increases the caloric intake of teens. Second, it provided more evidence for the unique impact of SR on adolescents’ perceptions and intake of foods that are generally regarded as sweets or desserts, such as ice cream, cake, and candy. Finally, it took an important step in the construction of a mechanistic causal model by which SR might influence dietary choices, suggesting that SR may increase the subjective appeal of already attractive sweet/dessert foods.

To our knowledge, this study is the first to investigate food-appeal in sleep-restricted adolescents. In adults, SR particularly increases intake of carbohydrates and fat [12,13], and cravings for calorie-dense foods [9], suggesting sleep deprivation makes these foods more rewarding. Consistent with this, adult functional neuroimaging studies show that pictures of appealing foods trigger a greater response in reward-relevant brain regions after SR than after a period of full rest [14]. These reward-relevant brain regions also are abnormally responsive to high-calorie foods among people with obesity [15]. Beyond promoting obesity, a diet of high-sugar foods has been independently linked to several chronic health conditions (e.g., metabolic syndrome, diabetes, heart disease) [16].

The proposed shift in hedonic value of sweets/desserts does not exclude other mechanisms by which SR might impact diet (e.g., circadian factors, disinhibition), an area ripe for additional work. We recommend future research also overcome several limitations of this study, including our limited outcome measures, measurement exclusively after 5 nights of SR (as opposed to examining change as sleep debt accumulates), small sample, and lack of assessment of participants’ activity level. Additionally, our study did not take into account female menstrual cycle; while sleep timing and composition show relatively little fluctuation across the menstrual cycle in healthy women [17], increases in caloric and macronutrient intake have been found during the midluteal phase [18]. Our study also used a relatively short “washout” period (2 days), though it is reassuring to note that we found no evidence of carryover effects across conditions, and similar experimental work has found that 2 days is sufficient to eliminate the effects of partial sleep restriction on dietary intake in adults [19]. Importantly, our use of a randomized cross-over design helped ensure that neither menstrual cycle nor length of washout would confound results, though both could affect error variance. Lastly, more sensitive measurements of hunger integrating both subjective and physiological measures would be helpful.

If future research corroborates present findings, the potential public health impact could be impressive. As biological and social factors push bedtimes later, teens’ schools start earlier, leading them to average 2-hours less sleep on school nights than recommended [6]. Because adolescents establish enduring dietary patterns [7], and because adolescent obesity is highly persistent [20], the effects of SR could have life-long consequences, even if short sleep is limited to the high school years.

## Supporting Information

S1 PDF Beebe DW, Simon S, Summer S, Hemmer S, Strotman D, et al. (2013). Dietary intake following experimentally restricted sleep in adolescents. Sleep 36: 827–834.
(PDF)

S1 File De-Identified Dataset. (ZIP)

S2 File Analyses Syntax. (ZIP)
