# Supplementary material for: Sweet/Dessert Foods Are More Appealing to Adolescents after Sleep Restriction
Source: PLoS One. 2015 Feb 23;10(2):e0115434. doi: 10.1371/journal.pone.0115434 (PMC4338308; doi:10.1371/journal.pone.0115434)
Supplement: S1 PDF — Dietary intake following experimentally restricted sleep in adolescents. Sleep 36: 827–834. (PDF) [file pone.0115434.s001.pdf]

http://dx.doi.org/10.5665/sleep.2704

Dietary Intake Following Experimentally Restricted Sleep in Adolescents

Dean W. Beebe, PhD[1,2]; Stacey Simon, PhD[1]; Suzanne Summer, MS, RD[1]; Stephanie Hemmer, BA[1]; Daniel Strotman, BA[1]; Lawrence M. Dolan, MD[1,2]

[1]*Cincinnati Children's Hospital Medical Center, Cincinnati, OH;* [2]*Department of Pediatrics, University of Cincinnati College of Medicine, Cincinnati, OH*

Study Objective: To examine the relationship between sleep and dietary intake in adolescents using an experimental sleep restriction protocol.
Design: Randomized crossover sleep restriction-extension paradigm.
Setting: Sleep obtained and monitored at home, diet measured during an office visit.
Participants: Forty-one typically developing adolescents age 14-16 years.
Interventions: The 3-week protocol consisting of a baseline week designed to stabilize the circadian rhythm, followed randomly by 5 consecutive nights of sleep restriction (6.5 hours in bed Monday-Friday) versus healthy sleep duration (10 hours in bed), a 2-night washout period, and a 5-night crossover period.
Measurements: Sleep was monitored via actigraphy and teens completed validated 24-hour diet recall interviews following each experimental condition.
Results: Paired-sample *t*-tests examined differences between conditions for consumption of key macronutrients and choices from dietary categories. Compared with the healthy sleep condition, sleep-restricted adolescents' diets were characterized by higher glycemic index and glycemic load and a trend toward more calories and carbohydrates, with no differences in fat or protein consumption. Exploratory analyses revealed the consumption of significantly more desserts and sweets during sleep restriction than healthy sleep.
Conclusions: Chronic sleep restriction during adolescence appears to cause increased consumption of foods with a high glycemic index, particularly desserts/sweets. The chronic sleep restriction common in adolescence may cause changes in dietary behaviors that increase risk of obesity and associated morbidity.
Keywords: Adolescence, glycemic index, glycemic load, obesity, pediatrics, sleep deprivation
Citation: Beebe DW; Simon S; Summer S; Hemmer S; Strotman D; Dolan LM. Dietary intake following experimentally restricted sleep in adolescents. *SLEEP* 2013;36(6):827-834.

INTRODUCTION

Obesity has become a well-recognized public health concern. Approximately one third of all children and adolescents and two thirds of adults in the United States are overweight or obese,[1,2] placing them at increased risk for physical and psychosocial morbidity. Existing prevention and intervention programs have failed to reverse the precipitous rise in obesity rates that has occurred in recent decades, fueling the search for new, potentially modifiable variables that can be targeted for obesity prevention or management.

Recent research has suggested that improved sleep duration may be one such target. In both children and adults, short sleep is associated with greater rates of obesity and prospectively predicts weight gain over time.[3-6] In adults, acute sleep deprivation increases caloric intake, due particularly to increased consumption of carbohydrates and fat.[7,8] Ratings of hunger and appetite are also greater after sleep restriction, with the largest cravings for sweet, salty, and high-starch foods.[9] Such foods may be particularly problematic if they are high in simple sugars (high glycemic index) consumed in large amounts (high glycemic load). Foods with a high glycemic index and glycemic load lead to dramatic fluctuations in serum glucose, and have been linked to the long-term development of several chronic diseases (e.g., diabetes, coronary heart disease, gallbladder disease, some cancers) and short-term increases in hunger.[10-12] Because sleep deprivation also alters glucose regulation,[13] it may be as important to determine what kinds of foods are linked to inadequate sleep as it is to determine how much food is consumed.

The potential link between inadequate sleep and dietary patterns takes on particular importance during adolescence due to a convergence of factors. Adolescence reflects the first time many individuals face chronic sleep restriction, as typical sleep duration plummets well below recommended levels on school nights.[14] At the same time, adolescents self-direct their food choices much more than do younger children[15] and dietary patterns that are established during adolescence tend to persist into adulthood.[16,17] Relatedly, adolescent obesity is a strong predictor of adult obesity[18] and imparts an increased risk of morbidity even for individuals who lose weight later.[19] If sleep duration influences dietary choices, the chronic sleep restriction that is endemic during adolescence, even if limited to the high school years, could have long-term effects on dietary patterns, body mass, and related morbidity.

Unfortunately, obesity prevention and treatment efforts have tended to be least effective for adolescents.[20,21] If a causal relationship is found between short sleep and unhealthy dietary choices during adolescence, this could pave the way for innovative approaches to both obesity intervention (e.g., sleep as a treatment target) and obesity prevention (e.g., delaying high school start times to lengthen adolescent sleep). The field has good correlational data; for each hour less sleep received by adolescents, there is an 80% increase in obesity risk.[22] What is needed now is complementary experimental work to

A commentary on this article appears in this issue on page 813.

Submitted for publication June, 2012
Submitted in final revised form October, 2012
Accepted for publication November, 2012
Address correspondence to: Dean W. Beebe, PhD, Division of Behavioral Medicine and Clinical Psychology, MLC 3015, Cincinnati Children's Hospital Medical Center, 3333 Burnet Avenue, Cincinnati, OH 45229; Tel: (513) 636-3489; Fax: (513) 636-7756; E-mail: dean.beebe@cchmc.org

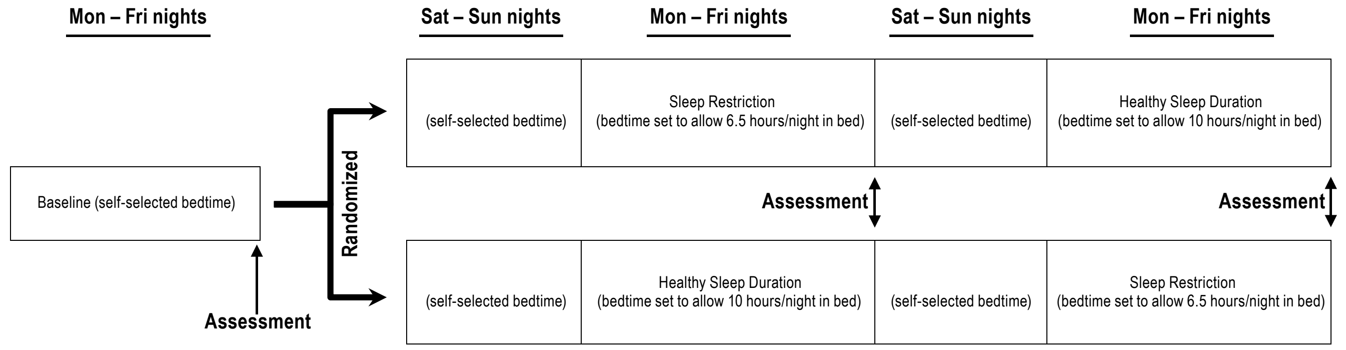

Figure 1—Schematic diagram of the sleep protocol. Teens were asked to maintain a constant wake time across all 3 weeks. They were allowed to self-select their bedtimes during the baseline week, as well as the Saturday and Sunday nights between conditions. On Monday-Friday nights during the experimental week, their bedtimes were modified to cause sleep restriction (sleep restriction; 6.5 h in bed per night) or allow healthy sleep duration (healthy sleep condition; 10 h in bed per night with lights out). Dietary assessments were conducted on the Saturday morning at the end of each experimental week, asking about intake throughout the previous day (Friday).

establish that short sleep, as opposed to a confounding factor, drives that association.

Adult findings provide a starting point, but several factors caution against simply extrapolating from adult sleep deprivation work. Adult studies have used sleep deprivation protocols that are more extreme (e.g., ≤ 4 h sleep/night) and/or last fewer days than the chronic sleep deficit that is common in teens.[7,8,23,24] In addition, rather than measuring dietary patterns under normal circumstances, most experimental deprivation studies of adults have measured subjective cravings[9] or food consumption in an artificial setting.[8,24] Consequently, the applicability of previous adult study findings to the daily experience of adolescents is uncertain. Developmental factors might also affect findings, as adolescents differ from adults in sleep architecture[25] and dietary needs.[26] Furthermore, adolescents and adults likely differ in how they perceive food. Adolescents receive unique messages via demographically targeted advertising campaigns[27] and tend not to use—or even understand—detailed nutrition information,[28] instead making food choices based on broader food groupings.[29]

The current study aimed to clarify whether multiple nights of sleep restriction, similar in length and severity to the experience of many adolescents on school nights, causes a change in the dietary patterns of adolescents who are otherwise engaged in normal daily activities. Based on previous studies of adults, we hypothesized that teens would consume more calories, carbohydrates, and fat, as well as foods with higher glycemic index and glycemic load, after several nights of restricted sleep than when well rested. We did not expect a difference in protein intake across conditions. To obtain a better sense of dietary changes in terms more familiar to adolescents (and to most adults), we also explored cross-condition differences in the amount of food consumed from particular dietary categories that have been implicated as important in the media (e.g., sweetened beverages, fast-food entrees, sweets/desserts).

METHODS

All procedures were approved and overseen by the Institutional Review Board at Cincinnati Children's Hospital Medical Center. Prior to participation, adolescents provided informed assent and their parents provided informed consent.

Participants

Healthy adolescents 14-16 y of age were recruited from flyers posted throughout a regional pediatric care network. Exclusion criteria included diagnosis of psychiatric disorder, history of neurologic illness or injury, body mass index (BMI) > 30, an Intelligence Quotient (IQ) < 70, regular high caffeine use (more than one coffee/energy drink or two caffeinated soft drinks per day), suspected recurrent illegal substance use, use of medication with known effects on sleep or daytime alertness, or obligations that would require bedtime later than 10:00pm or waking prior to 6:00am. Additionally, participants had to agree to refrain from driving for the sleep restriction portion of the study.

Procedures

The current study involved a new sample of participants, but followed procedures first outlined by Beebe and colleagues.[30] Each teen participant completed a 3-week experimental sleep manipulation protocol (Figure 1) during summer vacation so as not to interfere with school. Prior to participation, the adolescents and their parents were asked to determine the time at which the teens would need to awaken to arrive at the study location by 8:30am; this became the rise time during all 3 weeks. Week 1 (baseline) was designed to stabilize sleep patterns; on Monday-Friday nights the teens were allowed to self-select their bedtimes but asked to awaken at the predetermined rise time. During weeks 2 and 3, participants' prescribed bedtimes on Monday-Friday nights were systematically changed to allow 6.5 h in bed (sleep-restriction condition, SR) versus 10 h in bed with lights out and all phones and electronic screens off (healthy sleep duration, HS). The order of experimental conditions was counterbalanced across participants in a randomized crossover design.

Teens were allowed to self-select their bedtimes on Saturday and Sunday nights as a 2-night washout period between conditions. A 2-night washout was selected to balance the participant burden associated with extending study participation against what is known about recovery from sleep deprivation. Although we know of no data on how long it takes for dietary behaviors to normalize following sleep restriction, most research (in-

Table 1—Food categories, exemplars, and mean macronutrient characteristics per serving

Food category	Examples	Calories	Fat (g)	Carbohydrates (g)	Protein	Glycemic load	Glycemic index
Sweetened beverages	Nondiet soft drinks, sweetened fruit juices, and sports drinks	115	0.5	27.9	0.6	24.8	68.7
Unsweetened beverages	Water, artificially sweetened soft drinks or sports drinks	1	0.0	0.3	0.0	0.0	6.8
Fruits and vegetables	Unprocessed bananas, apples, or vegetable salad	53	0.8	11.9	1.2	7.1	71.9
Meat and eggs	Meat and eggs in isolation or lightly prepared	164	10.2	1.7	15.4	1.3	36.5
Processed snacks	Chips, crackers, french fries	163	8.0	21.1	2.3	18.7	97.3
Fast-food entrees	Hamburgers, pizza, sandwiches	325	15.6	30.1	15.6	26.1	94.1
Grains and starches	Bread, rice, pasta, cereal	181	4.1	30.0	6.0	24.7	89.9
Sweets and desserts	Candy, ice cream, milkshake	205	9.1	28.8	2.7	24.8	87.3

Macronutrient characteristics per serving were based upon USDA serving-size definitions, as applied to the actual food choices of teens in this study.

cluding the only relevant study of adolescents) suggests that neurobehavioral functioning normalizes after 1-2 nights of recovery sleep.[31-35] Rather than prescribe the sleep duration for the washout, we allowed teens to self-select their bedtimes because several stated in a pilot study that they thought they could better adhere to prescribed bedtimes during the experimental conditions if they had more control during the weekends.

All participant sleep occurred at home in their natural setting and was monitored via a daily sleep diary and objective actigraphy. Teens were also asked to refrain from napping and to limit caffeine intake to either one coffee or energy drink or two caffeinated sodas per day. Otherwise, they were not given any instructions regarding diet, and the dietary assessments comprised only about 15% of participants' activities during each weekly assessment session, with much more time devoted to assessments of cognitive, behavioral, and neurologic functioning (data not reported here).

Assessment visits took place on the Saturday mornings after each sleep condition. Sleep instructions were provided to both the teens and parents via phone (baseline week) or in person (experimental weeks). Families were provided with personalized written instructions and signs to remind them of the requested sleep schedule, and parents were encouraged to work with teens to promote successful execution of the schedule. Participants were compensated incrementally for each week of participation ($50, $75, and $100 for the 3 consecutive weeks).

Measures

Sleep Monitoring

Sleep was monitored for all nights of the study, including the baseline week, both experimental conditions, and weekends. Each participant wore on the nondominant wrist a Micro Motionlogger Sleep Watch (Ambulatory Monitoring Incorporated, NY), which collected data on movements (Zero-Crossing Mode) in 1-min bins. Because many teens were involved in sports and other activities that could damage the actigraphs, teens were allowed to remove the actigraph during the day, provided they put it on before bed, wore it all night, and took it off at least 5 min after rising. Sleep data were uploaded and reviewed with both the teen and parent during each Saturday assessment to verify accuracy, ensure removal of artifacts (e.g., failure to wear the actigraph during a portion of a night), and to promote a sense of accountability. Artifact-free data were run though a validated algorithm[36] and the following variables were averaged for each study participant across nights within the SR and HS conditions: nightly sleep onset, offset, and total sleep period (rise time minus sleep onset time).

Dietary Consumption

A 24-h diet recall was conducted each Saturday after both experimental sleep conditions using the United States Department of Agriculture (USDA) Multiple Pass Method. The 24-h dietary recall interview method has been validated in children against doubly labeled water[37] and weighed diet diaries.[38] Trained interviewers who were blind to experimental condition engaged participants in 15- to 20-min interviews to collect data regarding food items and amounts consumed over the previous 24-h periods. Participants were provided with instructions and handouts to assist them with estimating portion sizes during the interview. The Nutrition Data Systems for Research software (Nutrition Coordinating Center, University of Minnesota, Minneapolis, MN), which contains a comprehensive profile of more than 150 nutrients and other relevant data for more than 18,000 foods and 160,000 food variants, was then used to calculate nutritional information on foods consumed in each 24-h period. For primary analyses, we examined total calories (kilocalories); total grams of fat, carbohydrate, and protein; overall glycemic index; and overall glycemic load.

Because such nutritional data can be difficult for adolescents (and many adults) to translate into specific food choices, food choices were classified for exploratory analyses into discrete con-

ceptually driven categories (Table 1). Each food was assigned to a single category, although meals typically included multiple foods that fell in different categories (e.g., a fast-food entrée and a sweetened soda). For analyses, we focused on the most common categories consumed: sweetened sodas and fruit beverages, diet and unsweetened beverages, fruits and vegetables, meat and eggs, processed snacks, fast-food entrees, grains and starches, and sweets and desserts. Examples of foods and typical macronutrient features of each category are presented in Table 1. For each 24-h interview, we used USDA serving-size definitions to calculate the number of servings consumed by each teen from each category.

Descriptive Information

Anthropometric data were acquired at the time of the baseline evaluation using a calibrated scale and stadiometer, and height and weight were converted to age- and sex-adjusted BMI z-scores per 2000 US Centers for Disease Control and Prevention (CDC) tables. Family background and demographic information were obtained via parent questionnaire. Self-report questionnaires also queried adolescents' typical sleep duration on school nights and nonschool nights (e.g., weekends, holidays).

Analytic Strategy

Before analysis, each outcome variable underwent inspection for outliers, and participants were excluded from analyses if their dietary data were outliers or if they were nonadherent to the sleep protocol (operationalized as < 60 min difference in sleep duration across conditions). Preliminary analyses using general linear modeling indicated that none of our primary or exploratory outcome variables were significantly affected by an interaction of the experimental manipulation with teen sex or ethnicity (dichotomized as white-nonwhite).

The potential for carryover effects was statistically tested by entering as predictors the order in which the two sleep conditions were presented (order) and the interaction between order and the manipulation; a significant interaction term would indicate a carryover effect. Across all of our dietary and sleep duration outcome measures, the manipulation did not interact with the order in which the experimental conditions occurred (all $P > 0.05$). Thus, the effect of the sleep manipulation did not seem to vary based on whether HS or SR came first.

This lack of carryover effect allowed us to simplify our dietary analyses to paired-sample *t*-tests comparing participants across the SR and HS conditions. The directional (one-tailed) primary hypothesis was that, compared with the HS condition, during the SR condition participants would consume more total calories and greater grams of fat and carbohydrates, and also consume foods with a higher glycemic index and overall glycemic load. Paired-sample *t*-tests were also used for exploratory (two-tailed) analyses on the number of servings consumed within each dietary category across sleep conditions. Although some raw data distributions were skewed, we chose the *t*-test over a less powerful nonparametric statistic because the distributions of cross-condition change scores (upon which the paired-sample *t*-test is based) were roughly normal.

Power Analysis

We *a priori* estimated that we would find a small to moderate effect size of $d = 0.35$ for primary analyses based upon Cappuccio and colleagues'[6] pooled effect size across seven epidemiologic studies of the association between short sleep and weight in children. This estimate was chosen over effects reported from adult experimental studies because such studies used more extreme (and unsustainable) sleep curtailment schedules. With $d = 0.35$ and a paired-sample *t*-test, a sample size of 40 would achieve > 80% power for our primary analyses, without correction for multiple comparisons.

RESULTS

Sample Description

Of the 64 participants who consented to the study, 10 were nonadherent to the waking regimen during the baseline week and consequently were not randomized. Four randomized participants subsequently dropped out, and data from nine teens were excluded from further analyses because of nonadherence to the sleep protocol ($n = 4$) or daily intake exceeding 4,000 calories ($n = 5$), which was > 2.5 standard deviations (SD) above the rest of the sample for either condition. The final sample of 41 adolescents (59% female, 54% Caucasian, 37% African-American) had a mean age of 15.3 y (SD = 0.7), median parental education of 14 y (range = 7-20+), and median family income between $50,000-$60,000 US dollars (range = < $20,000 - > $150,000). Mean self-reported typical sleep duration was 9.3 h (SD = 1.4) on nonschool nights and 7.1 h (SD = 1.2) on school nights; 59% reported sleeping ≤ 7 h and 20% reported sleeping ≤ 6 h on typical school nights. Although we excluded for marked obesity (BMI > 30), the sample trended heavier than the 2000 CDC norms, consistent with a national and regional drift in body mass. The sample mean BMI was 23.4 (SD = 4.0) and mean BMI z-score was 0.7 (SD = 0.8); no participant fell below the fifth percentile, 66% fell in the 5th-85th percentile, 17% in the 85th-95th percentiles, and 17% above the 95th percentile. This demographic and BMI distribution approximates that of the general adolescent population in Cincinnati/Hamilton County.[39,40]

Manipulation Check

The actigraph units failed 5% of the weeks, apparently at random. This resulted in missing data for four participants, whose adherence to the sleep regimen was confirmed via parent and self-report. For the remaining 37 adolescents, Figure 2 displays actigraph-based estimates of sleep onset, offset, and sleep duration for the baseline condition, the weekends that preceded each experimental condition, and the SR and HS conditions. Not surprisingly, general linear modeling found significant differences in sleep duration across those periods ($P < 0.001$). The baseline and weekend sleep durations did not significantly differ ($P > 0.05$) and overall reflected a mean of 7.00 h (SD = 0.88), which was significantly shorter than during the HS condition (mean = 8.86 h, SD = 0.89, $P < 0.001$) and longer than during the SR condition (mean = 6.33 h, SD = 0.52, $P = 0.001$). Consistent with Beebe et al.,[30] the adolescents averaged 2.53 hours (SD = 0.68) more sleep during the HS condition than the SR condition ($P < 0.001$). Per the experimental instructions, that difference was due exclusively to a later sleep onset during SR compared with HS (mean sleep onset = 12:50am versus 10:21pm, $P < 0.001$), whereas waking times did not dif-

fer across the SR and HS conditions (mean wake time = 7:08am versus 7:11am, P = 0.561).

In exploratory analyses, participants' self-reported habitual sleep duration on school nights significantly correlated with their pooled sleep duration during the baseline and washout periods (r = 0.54, P = 0.001), but not length of sleep during the SR (r = 0.15, P = 0.38) or HS (r = 0.06, P = 0.73) conditions. None of the study sleep periods significantly correlated with self-reported habitual sleep duration on nonschool nights (P > 0.15), and neither school night nor nonschool night sleep duration correlated with the size of the gap in sleep duration between the HS and SR conditions (P > 0.70). In other words, habitual sleep duration did not appear to affect sleep behaviors during the 2 experimental weeks. However, habitual sleep duration on school nights significantly predicted sleep during the baseline and washout periods, when the adolescents were allowed to self-select their bedtimes, but had to awaken on time in the morning (similar to school nights).

Primary Analyses (Macronutrients)

Table 2 summarizes macronutrient findings across the two conditions. The teens' intake of fat and protein did not significantly differ across the experimental conditions (P > 0.15). However, compared with the HS condition, during sleep restriction the teens consumed foods with a significantly higher glycemic index (P = 0.046) and glycemic load (P = 0.037), and showed trends toward greater consumption of total calories (P = 0.098) and carbohydrates (P = 0.070).

Exploratory Analyses (Food Categories)

Table 3 summarizes the number of servings of food consumed in each qualitative category. Exploratory analyses revealed a significant difference in consumption of sweets and desserts across sleep conditions. On average, the teens consumed a full serving more sweets and desserts during the final day of the SR condition than during the equivalent day of HS (P = 0.016). No differences were noted between conditions for the other dietary categories (P > 0.10).

DISCUSSION

Compared with dietary intake following several nights of nearly 9 h of sleep, adolescents' diets after several nights of sleep restriction were characterized by higher glycemic index and glycemic load, as well as a trend toward greater consumption of calories and carbohydrates. Exploratory analyses suggested a particularly increased consumption of desserts and sweets during sleep restriction. Although this study warrants replication and extension, results are consistent with prior epidemiologic studies that show an association between short sleep and both concurrent weight and heightened weight gain over time.[3-6] Complementing prior findings, our experimental data provide unprecedented evidence that the chronic sleep restriction common in adolescence may cause changes in dietary behaviors that substantially increase the risks of obesity and associated morbidity.

Effect sizes were modest and somewhat smaller than expected based on the pooled association between childhood sleep duration and weight in large correlational studies.[6] However, prior

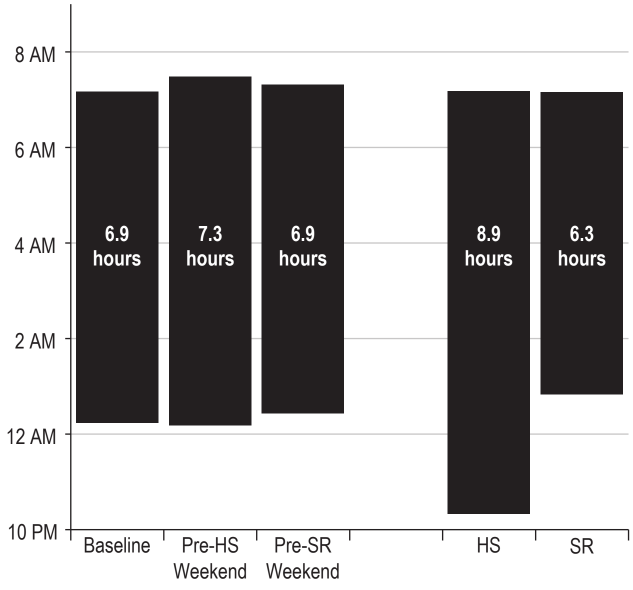

Figure 2—Average sleep patterns, as estimated by actigraphy, for the baseline condition, the weekends that preceded each experimental condition, and each experimental condition (SR, sleep restriction; HS, healthy sleep condition). Mean sleep onset time is marked by the bottom of each bar, and sleep offset by the top of each bar. Mean sleep duration is printed within each bar. The baseline and weekend sleep durations did not significantly differ (P > 0.05) but collectively were significantly shorter than the HS condition (P < 0.001) and longer than the SR condition (P = 0.001). The adolescents averaged 2.53 h (SD = 0.68) more sleep during the HS condition than the SR condition (P < 0.001) due to changes in sleep onset (P < 0.001) without differences in wake times (P = 0.561).

Table 2—Daily macronutrient consumption across sleep conditions

	Sleep restriction	Healthy sleep	*t* (df = 40)	P	Effect size (d)
Calories/energy (kcal)	1,953 ± 788	1,796 ± 724	1.32	0.098	0.21
Fat (grams)	76.5 ± 35.6	71.1 ± 38.3	0.94	0.175	0.15
Carbohydrates (grams)	257.2 ± 117.0	229.6 ± 97.0	1.51	0.070	0.24
Protein (grams)	64.4 ± 28.7	64.2 ± 31.5	0.05	0.481	0.01
Glycemic index	89.6 ± 6.0	87.1 ± 8.6	1.73	0.045	0.27
Glycemic load	221.3 ± 109.1	190.6 ± 86.3	1.84	0.037	0.29

Data are presented as mean ± standard deviation. Significance P values reflect directional one-tailed hypothesis tests. kcal = kilocalories, conventionally shortened to calories.

Table 3—Daily number of servings consumed in each food category across sleep conditions

	Sleep restriction	Healthy sleep	*t* (df = 40)	P	Effect size (d)
Sweetened beverages	2.06 ± 2.22	1.56 ± 2.22	1.40	0.170	0.22
Unsweetened beverages	2.32 ± 2.12	3.16 ± 3.16	-1.60	0.118	-0.25
Fruits and vegetables	0.53 ± 1.13	0.45 ± 1.09	0.63	0.758	0.05
Meat and eggs	1.07 ± 1.13	1.02 ± 1.20	0.49	0.773	0.05
Processed snacks	1.36 ± 1.54	1.47 ± 1.65	-0.36	0.724	-0.06
Fast-food entrees	1.15 ±1.32	1.21 ± 1.48	-0.20	0.841	-0.03
Grains and starches	1.98 ± 1.66	1.69 ± 1.40	1.20	0.237	0.19
Sweets and desserts	1.89 ± 2.35	0.82 ± 0.99	2.52	0.016	0.39

Data are presented as mean ± standard deviation. Significance P values reflect nondirectional/exploratory two-tailed tests.

findings have varied substantially, and most adult epidemiologic work has reported effect sizes similar to the experimental effects found here.[6] Importantly, if current findings prove to be reliable in larger-scale work, they are potentially substantial if multiplied over time. For example, although the number of calories consumed during sleep restriction was only 9% above that consumed during the healthy sleep condition, the cumulative surplus across the school days in an academic year could exceed 28,000 calories, or approximately 5-10 excess pounds per year if there is no compensatory change in caloric expenditure.

Caloric intake aside, the chronic consumption of foods with a high glycemic index and glycemic load has been linked prospectively to the development of diabetes, coronary heart disease, gallbladder disease, and certain cancers in adults.[10] Less is known about the short-term health effect on children, but consumption of foods with a high glycemic load appears to increase the risk of metabolic syndrome in teens.[41] Diets comprising foods with a high glycemic index may also make weight management more difficult. Foods with a high glycemic index spur a rapid homeostatic response to stabilize serum glucose levels, which some data suggest may hasten the subsequent experience of hunger and overeating.[11,12] Even when caloric intake is identical, a diet that limits high-glycemic foods results in better preservation of adults' resting energy metabolism during weight loss than a diet that focuses on fat intake.[42] We are not aware of comparable work in adolescents, but note that a low-glycemic diet was better tolerated than calorically similar low-carbohydrate or traditional portion-control diets in a recent randomized treatment trial with overweight youth.[43] Even if only a small shift in health risk is associated with the dietary effects of chronic sleep restriction, the result could still be substantial on a population level. The duration of sleep obtained during our sleep restriction condition—6.3 h/night—is experienced on school nights by more than one sixth of adolescents in the US,[14] or more than five million teens.

Although previous research has been fairly consistent in relating sleep durations less than 7 h with excess body mass, associations between sleep and specific macronutrients have varied. Beebe and colleagues[44] found that severity of sleep disordered breathing in obese individuals age 10-16 y predicted increased preference for foods high in calories, fat, and carbohydrates. Weiss and colleagues[45] associated shorter actigraphy-measured weekday sleep duration with greater intake of calories, more of which came from fat and less from carbohydrates. Other studies have confirmed associations between short sleep and consumption of calorie-dense foods in children,[46] but have not allowed for examination of macronutrient content. The adult experimental literature similarly supports a causal link between inadequate sleep and an unhealthy diet, but some authors have reported increases in carbohydrate-rich foods[7,24] and others have emphasized changes in dietary fat.[8,23] Prior experimental studies have not specifically addressed glycemic index or glycemic load, though adult researchers have implicated short sleep in the dysregulation of glucose metabolism.[47] This poor glucose regulation, when combined with our finding of a selective increase in foods that are high in glycemic index and load, raises the alarming possibility that chronic sleep restriction may be a tipping point in the development of diabetes in vulnerable adolescents.

To some degree, the variability in macronutrient findings across studies may relate to methodologic factors, including sample composition, duration and intensity of sleep restriction, and how diet was measured. Given that several experimental studies of adults focused on sleep and diet in artificial laboratory settings,[8,9,24] it is encouraging to note the multiple points of convergence with our findings of typical free-living eating patterns after sleep restriction in the home environment. Similarly, it is encouraging to note that the results of studies of short-term, fairly intense sleep deprivation in adults[7,8,23,24] converge with findings from our longer-term, more realistic sleep restriction in adolescents: sleep restriction seems to cause dietary changes.

The mechanisms that might underlie such a causal relationship remain speculative. Individuals who are awake longer have more eating opportunities, but that cannot account for the selective consumption of sweet foods during sleep restriction. Sweet foods are often readily available for consumption, but so are less-sweet foods that showed no effect of our sleep manipulation. Although extending beyond the current data, physiologic and neurobehavioral mechanisms may be more relevant. Levels of appetite-suppressing leptin and hunger-promoting ghrelin appear to be influenced by sleep duration, and short sleep may further decrease physiologic sensitivity to satiety cues.[48] In addition, ghrelin appears to activate a cholinergic-dopaminergic reward pathway[49] and sweets may be particularly rewarding because simple sugars cause dopamine release in reward centers.[50] In adults, even viewing pictures of food items,[51] especially calorie-dense foods,[52] triggers a greater response in these reward circuits after sleep deprivation than after a period of full

rest. Given that short sleep in adolescents is otherwise associated with diminished sensitivity of reward circuits,[53] sleep-deprived teens may amplify reward-seeking behaviors, including consumption of sweet foods.

Additional research is needed to test these putative mechanisms and to overcome the several limitations of the current study. A key limitation is that dietary intake was obtained via an interview that has been validated but is subject to reporting inaccuracies.[54] The fact that dietary data were obtained on only a single 24-h period toward the end of each sleep condition further raises concerns about the reliability of the dietary data. Diminished reliability tends to attenuate results rather than spuriously inflate them, but in future work we advise more frequent sampling of dietary data, which would also allow for examination of changes in diet over time, as sleep restriction accrues a deepening sleep debt.

Our sample is larger than any previously published experimental sleep-diet study and this study maximized statistical power by using a within-subject design and conceptually guided directional (one-tailed) statistical tests. However, the study may nevertheless have been underpowered to detect very small effects, and could not incorporate rigorous controls of Type I (false discovery) error without abandoning statistical power. In light of ongoing debate in the field about when and how to best use rigorous Type I error controls (e.g., Bonferroni corrections) and directional versus non-directional tests, current results are best viewed as promising, but requiring more definitive larger-sample follow-up. We also recommend specific examination in obese youth who are actively changing their diet, to check whether sleep duration affects their success. Finally, future work should assess participants' activity level, which we did not quantify in part because many teens told us that they removed their actigraph when engaged in sports or physical activities in which it might have been broken.

Balancing these limitations, the study reflects a significant advance over prior work in several respects. To our knowledge, it is the first study to examine dietary intake following an experimental sleep restriction protocol in adolescents, providing important evidence of causation in a uniquely relevant developmental period. It is also the first to create a level and duration of sleep restriction that is similar to that experienced by many adolescents on a regular basis, and one of few experimental sleep studies to measure diet in participants' natural environment, thereby helping to bridge the gap between current findings and complementary epidemiologic work. Most importantly, current findings dovetail with and expand on a growing evidence base that chronic sleep restriction during adolescence is far from benign, with consequences ranging from neurobehavioral deficits[44] to dietary changes that can have long-term health implications. Adolescents tend to have poorer dietary quality than other age groups[55]; this tendency appears to be amplified by sleep restriction.

ACKNOWLEDGMENTS

This study was funded by National Institutes of Health (R01 HL092149, UL1 RR026314). Research activities were conducted exclusively at Cincinnati Children's Hospital Medical Center.

DISCLOSURE STATEMENT

This was not an industry supported study. The authors have indicated no financial conflicts of interest.

REFERENCES

1. Flegal KM, Carroll MD, Kit BK, Ogden CL. Prevalence of obesity and trends in the distribution of body mass index among US adults, 1999-2010. JAMA 2012;307:491-7.
2. Ogden CL, Carroll MD, Kit BK, Flegal KM. Prevalence of obesity and trends in body mass index among US children and adolescents, 1999-2010. JAMA 2012;307:483-90.
3. Seegers V, Petit D, Falissard B, et al. Short sleep duration and body mass index: a prospective longitudinal study in preadolescence. Am J Epidemiol 2011;173:621-9.
4. O'Dea JA, Dibley MJ, Rankin NM. Low sleep and low socioeconomic status predict high body mass index: a 4-year longitudinal study of Australian schoolchildren. Pediatr Obes 2012;7:295-303.
5. Patel SR, Hu FB. Short sleep duration and weight gain: a systematic review. Obesity (Silver Spring) 2008;16:643-53.
6. Cappuccio FP, Taggart FM, Kandala NB, et al. Meta-analysis of short sleep duration and obesity in children and adults. Sleep 2008;31:619-26.
7. Bosy-Westphal A, Hinrichs S, Jauch-Chara K, et al. Influence of partial sleep deprivation on energy balance and insulin sensitivity in healthy women. Obes Facts 2008;1:266-73.
8. Brondel L, Romer MA, Nougues PM, Touyarou P, Davenne D. Acute partial sleep deprivation increases food intake in healthy men. Am J Clin Nutr 2010;91:1550-9.
9. Spiegel K, Tasali E, Penev P, Van Cauter E. Brief communication: sleep curtailment in healthy young men is associated with decreased leptin levels, elevated ghrelin levels, and increased hunger and appetite. Ann Intern Med 2004;141:846-50.
10. Barclay AW, Petocz P, McMillan-Price J, et al. Glycemic index, glycemic load, and chronic disease risk--a meta-analysis of observational studies. Am J Clin Nutr 2008;87:627-37.
11. Ludwig DS, Majzoub JA, Al-Zahrani A, Dallal GE, Blanco I, Roberts SB. High glycemic index foods, overeating, and obesity. Pediatrics 1999;103:E26.
12. Roberts SB. High-glycemic index foods, hunger, and obesity: is there a connection? Nutr Rev 2000;58:163-9.
13. Morselli LL, Guyon A, Spiegel K. Sleep and metabolic function. Pflugers Arch 2012;463:139-60.
14. Wolfson AR, Carskadon MA. Sleep schedules and daytime functioning in adolescents. Child Dev 1998;69:875-87.
15. Gillman MW, Rifas-Shiman SL, Frazier AL, et al. Family dinner and diet quality among older children and adolescents. Arch Fam Med 2000;9:235-40.
16. Larson NI, Neumark-Sztainer D, Story M. Weight control behaviors and dietary intake among adolescents and young adults: longitudinal findings from Project EAT. J Am Diet Assoc 2009;109:1869-77.
17. Arcan C, Neumark-Sztainer D, Hannan P, van den Berg P, Story M, Larson N. Parental eating behaviours, home food environment and adolescent intakes of fruits, vegetables and dairy foods: longitudinal findings from Project EAT. Public Health Nutr 2007;10:1257-65.
18. Craigie AM, Matthews JN, Rugg-Gunn AJ, Lake AA, Mathers JC, Adamson AJ. Raised adolescent body mass index predicts the development of adiposity and a central distribution of body fat in adulthood: a longitudinal study. Obes Facts 2009;2:150-6.
19. Dietz WH. Health consequences of obesity in youth: childhood predictors of adult disease. Pediatrics 1998;101:518-25.
20. Steele RG, Aylward BS, Jensen CD, Cushing CC, Davis AM, Bovaird JA. Comparison of a family-based group intervention for youths with obesity to a brief individual family intervention: a practical clinical trial of positively fit. J Pediatr Psychol 2012;37:53-63.
21. Waters E, de Silva-Sanigorski A, Hall BJ, et al. Interventions for preventing obesity in children. Cochrane Database Syst Rev 2011(12):CD001871.
22. Gupta NK, Mueller WH, Chan W, Meininger JC. Is obesity associated with poor sleep quality in adolescents? Am J Hum Biol 2002;14:762-8.
23. St-Onge MP, Roberts AL, Chen J, et al. Short sleep duration increases energy intakes but does not change energy expenditure in normal-weight individuals. Am J Clin Nutr 2011;94:410-6.
24. Nedeltcheva AV, Kilkus JM, Imperial J, Kasza K, Schoeller DA, Penev PD. Sleep curtailment is accompanied by increased intake of calories from snacks. Am J Clin Nutr 2009;89:126-33.
25. Carskadon MA. Sleep in adolescents: the perfect storm. Pediatr Clin North Am 2011;58:637-47.

26. Stang J, Story M, eds. Guidelines for adolescent nutrition services. Minneapolis, MN: Center for Leadership, Education and Training in Maternal and Child Nutrition, Division of Epidemiology and Community Health, School of Public Health, University of Minnesota, 2005.
27. Strasburger VC. Children, adolescents, obesity, and the media. Pediatrics 2011;128:201-8.
28. Campos S, Doxey J, Hammond D. Nutrition labels on pre-packaged foods: a systematic review. Public Health Nutr 2011;14:1496-506.
29. McCullum C, Achterberg CL. Food shopping and label use behavior among high school-aged adolescents. Adolescence 1997;32:181-97.
30. Beebe DW, Fallone G, Godiwala N, et al. Feasibility and behavioral effects of an at-home multi-night sleep restriction protocol for adolescents. J Child Psychol Psychiatry 2008;49:915-23.
31. Drummond SP, Paulus MP, Tapert SF. Effects of two nights sleep deprivation and two nights recovery sleep on response inhibition. J Sleep Res 2006;15:261-5.
32. Jay SM, Lamond N, Ferguson SA, Dorrian J, Jones CB, Dawson D. The characteristics of recovery sleep when recovery opportunity is restricted. Sleep 2007;30:353-60.
33. Lamond N, Jay SM, Dorrian J, Ferguson SA, Jones C, Dawson D. The dynamics of neurobehavioural recovery following sleep loss. J Sleep Res 2007;16:33-41.
34. Dinges DF, Pack F, Williams K, et al. Cumulative sleepiness, mood disturbance, and psychomotor vigilance performance decrements during a week of sleep restricted to 4-5 hours per night. Sleep 1997;20:267-77.
35. Carskadon MA, Harvey K, Dement WC. Sleep loss in young adolescents. Sleep 1981;4:299-312.
36. Sadeh A, Sharkey KM, Carskadon MA. Activity-based sleep-wake identification: an empirical test of methodological issues. Sleep 1994;17:201-7.
37. Johnson RK, Driscoll P, Goran MI. Comparison of multiple-pass 24-hour recall estimates of energy intake with total energy expenditure determined by the doubly labeled water method in young children. J Am Diet Assoc 1996;96:1140-4.
38. Klesges RC, Klesges LM, Brown G, Frank GC. Validation of the 24-hour dietary recall in preschool children. J Am Diet Assoc 1987;87:1383-5.
39. U.S. Census Bureau. 2011 American Community Survey. 2011. Available from: http://www.census.gov/acs/www/. Accessed November 21, 2012.
40. James M. Anderson Center for Health Systems Excellence. BMI Measurements in Cincinnati Public School Students. 2012. Available from: http://www.cincinnatichildrens.org/service/j/anderson-center/community-population-health/obesity/. Accessed November 21, 2012.
41. O'Sullivan A, Gibney MJ, Brennan L. Dietary intake patterns are reflected in metabolomic profiles: potential role in dietary assessment studies. Am J Clin Nutr 2011;93:314-21.
42. Ebbeling CB, Swain JF, Feldman HA, et al. Effects of dietary composition on energy expenditure during weight-loss maintenance. JAMA 2012;307:2627-34.
43. Kirk S, Brehm B, Saelens BE, et al. Role of carbohydrate modification in weight management among obese children: a randomized clinical trial. J Pediatr 2012;161:320-7.
44. Beebe DW, Miller N, Kirk S, Daniels SR, Amin R. The association between obstructive sleep apnea and dietary choices among obese individuals during middle to late childhood. Sleep Med 2011;12:797-9.
45. Weiss A, Xu F, Storfer-Isser A, Thomas A, Ievers-Landis CE, Redline S. The association of sleep duration with adolescents' fat and carbohydrate consumption. Sleep 2010;33:1201-9.
46. Westerlund L, Ray C, Roos E. Associations between sleeping habits and food consumption patterns among 10-11-year-old children in Finland. Br J Nutr 2009;102:1531-7.
47. Spiegel K, Tasali E, Leproult R, Van Cauter E. Effects of poor and short sleep on glucose metabolism and obesity risk. Nat Rev Endocrinol 2009;5:253-61.
48. Knutson KL, Van Cauter E. Associations between sleep loss and increased risk of obesity and diabetes. Ann N Y Acad Sci 2008;1129:287-304.
49. Landgren S, Simms JA, Thelle DS, et al. The ghrelin signalling system is involved in the consumption of sweets. PLoS One 2011;6:e18170.
50. Thornley S, Russell B, Kydd R. Carbohydrate reward and psychosis: an explanation for neuroleptic induced weight gain and path to improved mental health? Curr Neuropharmacol 2011;9:370-5.
51. St-Onge MP, McReynolds A, Trivedi ZB, Roberts AL, Sy M, Hirsch J. Sleep restriction leads to increased activation of brain regions sensitive to food stimuli. Am J Clin Nutr 2012;95:818-24.
52. Benedict C, Brooks SJ, O'Daly OG, et al. Acute sleep deprivation enhances the brain's response to hedonic food stimuli: an fMRI study. J Clin Endocrinol Metab 2012;97:E443-7.
53. Holm SM, Forbes EE, Ryan ND, Phillips ML, Tarr JA, Dahl RE. Reward-related brain function and sleep in pre/early pubertal and mid/late pubertal adolescents. J Adolesc Health 2009;45:326-34.
54. Burrows TL, Martin RJ, Collins CE. A systematic review of the validity of dietary assessment methods in children when compared with the method of doubly labeled water. J Am Diet Assoc 2010;110:1501-10.
55. Goodwin DK, Knolb LK, Eddy JM, Fitzhugh EC, Kendrick O, Donohue RE. Sociodemographic correlates of overall dietary intake of US adolescents. Nutrition Res 2006;26:105-10.
